# Pharmacogenetics and Forensic Toxicology: A New Step towards a Multidisciplinary Approach

**DOI:** 10.3390/toxics9110292

**Published:** 2021-11-04

**Authors:** Nunzio Di Nunno, Massimiliano Esposito, Antonina Argo, Monica Salerno, Francesco Sessa

**Affiliations:** 1 Department of History, Society and Studies on Humanity, University of Salento, 73100 Lecce, Italy; nunzio.dinunno@unisalento.it; 2 Department of Medical, Surgical and Advanced Technologies “G.F. Ingrassia”, University of Catania, 95121 Catania, Italy; massimiliano.esposito91@gmail.com; 3 Department of Health Promotion Sciences, Section of Legal Medicine, Maternal and Infant Care, Internal Medicine and Medical Specialties (PROMISE), University of Palermo, Via del Vespro, 129, 90127 Palermo, Italy; antonella.argo@unipa.it; 4 Department of Clinical and Experimental Medicine, University of Foggia, 71122 Foggia, Italy

**Keywords:** pharmacogenetics, forensic toxicology, multidisciplinary approach, molecular autopsy, drug-related death

## Abstract

Pharmacogenetics analyzes the individual behavior of DNA genes after the administration of a drug. Pharmacogenetic research has been implemented in recent years thanks to the improvement in genome sequencing techniques and molecular genetics. In addition to medical purposes, pharmacogenetics can constitute an important tool for clarifying the interpretation of toxicological data in post-mortem examinations, sometimes crucial for determining the cause and modality of death. The purpose of this systematic literature review is not only to raise awareness among the forensic community concerning pharmacogenetics, but also to provide a workflow for forensic toxicologists to follow in cases of unknown causes of death related to drug use/abuse. The scientific community is called on to work hard in order to supply evidence in forensic practice, demonstrating that this investigation could become an essential tool both in civil and forensic contexts. The following keywords were used for the search engine: (pharmacogenetics) AND (forensic toxicology); (pharmacogenetics) AND (post-mortem); (pharmacogenetics) AND (forensic science); and (pharmacogenetics) AND (autopsy). A total of 125 articles were collected. Of these, 29 articles were included in this systematic review. A total of 75% of the included studies were original articles (*n* = 21) and 25% were case reports (*n* = 7). A total of 78% (*n* = 22) of the studies involved deceased people for whom a complete autopsy was performed, while 22% (*n* = 6) involved people in good health who were given a drug with a subsequent pharmacogenetic study. The most studied drugs were opioids (codeine, morphine, and methadone), followed by antidepressants (tricyclic antidepressants and venlafaxine). Furthermore, all studies highlighted the importance of a pharmacogenetics study in drug-related deaths, especially in cases of non-overdose of drugs of abuse. This study highlights the importance of forensic pharmacogenetics, a field of toxicology still not fully understood, which is of great help in cases of sudden death, deaths from overdose, deaths after the administration of a drug, and also in cases of complaint of medical malpractice.

## 1. Introduction

Forensic science is a multidisciplinary science that uses the principles of different fields, such as chemistry, physics, and biology, in order to supply the so-called “weight of evidence” [1,2,3,4,5,6].

In this multidisciplinary context, the forensic toxicology laboratory aims to identify and quantify the presence of drugs and other chemical substances in biological fluids collected during autopsy [7,8,9]. Moreover, the toxicological investigation could be performed for medico-legal purposes (i.e., drug and alcohol tests for commercial driver licensing or involving occupational medicine due to the consequences of workers exposed to toxic substances) [10,11].

In forensic toxicology, it is essential to understand the pivotal role of pharmacogenetics (the study of how genetic variations give rise to differences in drug response), considering that pharmacokinetics and pharmacodynamics, and therefore the bioavailability of a drug, are conditioned by a genetic substrate. In forensic pathology, the diagnosis of the cause of death is guaranteed by the results of the crime scene investigation, anamnesis, autopsy, and toxicological data [12,13,14,15]. In this scenario, pharmacogenetics may be helpful to solve toxicological puzzles, especially in cases of suicides, accidents, and death by unknown causes. The possible influence of pharmacogenetics on drug metabolism should also be considered when interpreting the post-mortem concentration of a substance in body fluids or organs.

The term ‘pharmacogenetics’ is commonly used to describe the study of inter-individual variations in the DNA sequence that may affect the pharmacokinetics or pharmacodynamics of the drug. Indeed, these variations may influence genes encoding transporter proteins and enzymes that metabolize drugs, receptors, etc. [16]. The goal of pharmacogenetics is to predict the individual response to the drug and evaluate its effects. In clinical practice, pharmacogenetics is used to assess the ability of a patient, or a group of patients, to develop the desired effect or, conversely, to develop adverse effects [17,18]. Pharmacogenetic research has been implemented in recent years thanks to the improvement of genome sequencing techniques and molecular genetics. In medicine, pharmacogenetic research has two crucial functions: to identify disease-specific genes and gene products, and to identify allelic variants that influence the response to therapy [19].

Furthermore, pharmacogenetics may represent an important tool to clarify the interpretation of toxicological data in post-mortem examinations, sometimes crucial for determining the cause and modality of death. In fact, forensic pathologists are often implicated in deaths related to the use/abuse of drugs and narcotics [20]. For example, the CYP2D6 (Cytochrome P450 Family 2 Subfamily D Member 6) gene is involved in the metabolism of some drugs, such as morphine and its derivatives (codeine, tramadol, dihydrocodeine, oxycodone), and some CYP2D6 polymorphisms can reduce methadone clearance. Instead, CYP3A4 is involved in the metabolism of benzodiazepines and buprenorphine [21]. Thus, determining the presence of cytochrome inducing or suppressing mutations can provide answers in cases of death related to a suspected drug overdose [22].

Table 1 shows the main genetic polymorphisms of cytochromes involved in pharmacogenetics.

The purpose of this systematic literature review is not only to raise awareness among the forensic community regarding pharmacogenetics, but also to provide a workflow for forensic toxicologists to follow in cases of unknown causes of death related to drug use/abuse. The scientific community is called on to work hard in order to supply evidence in forensic practice, demonstrating that this investigation could become an essential tool both in civil and forensic contexts.

## 2. Materials and Methods

A systematic review was conducted.

SCOPUS was used as the search engine from 1 January 1980 to 1 August 2021 to evaluate the association between pharmacogenetics and forensic toxicology. The following keywords were used: (pharmacogenetics) AND (forensic toxicology); (pharmacogenetics) AND (post-mortem); (pharmacogenetics) AND (forensic sciences); and (pharmacogenetics) AND (autopsy).

### 2.1. Inclusion and Exclusion Criteria

The following exclusion criteria were used: (1) review, (2) articles not in English, (3) abstract, (4) poster, and (5) communications at conferences. The inclusion criteria were as follows: (1) Original Article, (2) Case Report, (3) Articles in English, (4) In Vivo Studies, and (5) In Vitro Studies.

### 2.2. Quality Assessment and Data Extraction

M.E. and F.S. initially evaluated all the articles, evaluating the title, the abstract, and the whole text. M.S., then, reanalyzed the articles chosen independently. In cases in which there were conflicting opinions regarding the articles, they were submitted to C.P.

### 2.3. Characteristics of Eligible Studies

**Figure 1:**
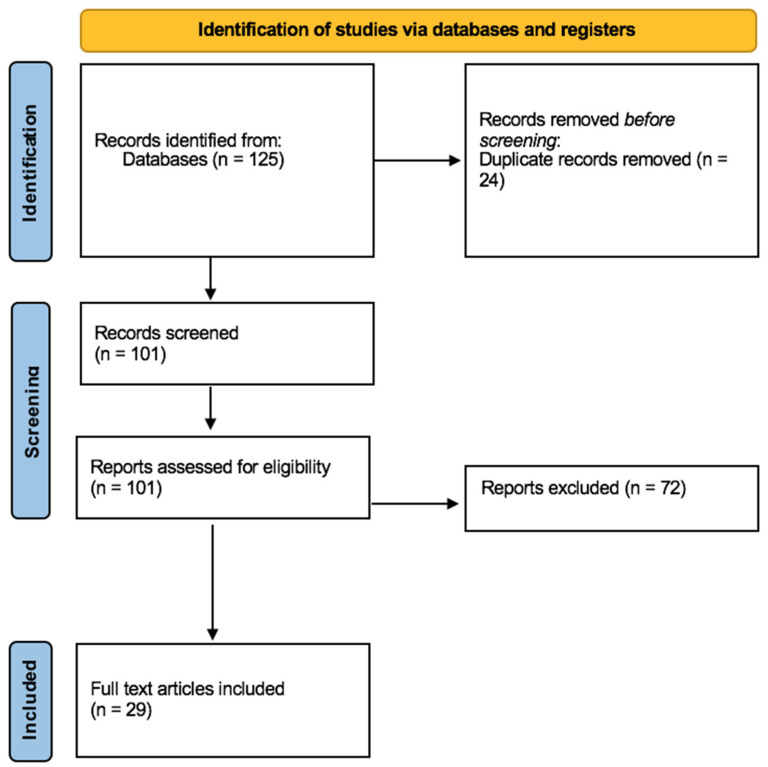
Flow diagram illustrating included and excluded studies in this systematic review.

A total of 125 articles were collected. Of these, 24 duplicates were removed. A total of 72 articles did not meet the inclusion criteria. In conclusion, 29 articles were included in the present systematic review (Figure 1).

## 3. Results

All selected studies are summarized in Table 2.

More that 75% of the included studies (*n* = 29) were original articles (*n* = 22), while about 25% were case reports (*n* = 7). In 22 of the included studies, a complete autopsy was performed, while about 20% (*n* = 6) concerned healthy people who had died after drug administration with a subsequent pharmacogenetic study. Opioids (codeine, morphine, and methadone) were used in most of the analyzed cases, followed by antidepressants (tricyclic antidepressants and venlafaxine). Only in one study ethylenediaminetetraacetic (EDTA) anticoagulant [26] and organophosphorus drugs [33] were administered [26,33,37]. A blood analysis was performed in all cases with one exception: in this case, a hair sample was used to analyze genetic polymorphism [31]. In one report, different post-mortem samples were analyzed, such as saliva, spleen, and buccal swabs [37]. The analysis of the techniques used to perform the toxicological investigation showed that gas chromatography–mass spectrometry (GC–MS) was the gold standard method, followed by liquid chromatography–mass spectrometry (LC–MS). Moreover, gas chromatography (GC) and high-performance liquid chromatography (HPLC) were used in two studies [35,41]. Only in one study was the toxicological investigation performed by a tandem mass spectrometer (UPLC–MS/MS) [27].

The most common method to analyze genetic polymorphisms was Polymerase Chain Reaction (PCR): the most investigated among the genetic polymorphisms was CYP2D6, followed by CYP2C19. Only two studies [47,49] were concerned with P-glycoprotein, and only one study referred to cytochrome UGT2B7 [40].

Analyzing all selected studies, the importance of pharmacogenetics as a pivotal tool in order to define the exact cause of death was clear, while for three studies [30,36,49] the cause of death remained unclear. Different genetic polymorphisms of the genes encoding cytochromes were investigated, determining changes in the bioavailability of the used drug and an increase in its toxicity. In several studies, it was reported that, although the drugs were administrated at the correct dosage, either due to the interaction with other drugs or due to the altered metabolization caused by these allelic variants, toxicity increased, causing the death of the subject [31,32,33,34,35,52,53,54,55,56,57,58]. In addition to the single nucleotide polymorphisms (SNPs) of the cytochrome genes, other genetic variations were investigated on the genes encoding for P-glycoprotein (P-gp). For example, Neuvonen A.M. et al. [47] analyzed the blood of 112 deceased people, clarifying a direct link between ABCB1 polymorphisms and the increase in mortality after digoxin treatment, suggesting a pivotal role for genotyping analysis.

Finally, all studies highlighted the importance of a pharmacogenetic study in drug-related deaths, especially in cases in which an overdose on illicit drugs, alcohol, prescription medications, and other kinds of substances was not found [19,25,26,27,28,29,30,31,32,33,34,35,36,37,38,39,40,41,42,43,44,45,46,47,48,49,50,51]. In these cases, the evaluation of different polymorphisms that could be related to drug assumption is strongly recommended [33]. The main findings of the genetic investigation in order to define the cause of death are summarized in Table 2.

## 4. Discussion

Forensic pharmacogenetics is a field of toxicology that studies and analyzes the genotypes and allelic variants of genes encoding enzymes involved in drug metabolism [53,54,55,56,57]. The results of the present review confirmed that one of the most studied genes is CYP2D6, which encodes a member of the cytochrome P450 superfamily of enzymes. Beause of the genetic polymorphisms of the CYP2D6 gene, there are four main phenotypes: ultra-rapid metabolisers (UM); extensive metabolisers (EM); poor metabolisers (PM), lacking functional enzymes due to defective or deleted the relative genes; and intermediate metabolisers (IM), carrying alleles that partially decrease enzyme activity [58]. A recent review [59] affirmed that 7% of people who died from an opioid overdose had an ultra-rapid phenotype with increased the toxic effect. As summarized in Table 1, the forensic interest in genotyping this cytochrome is related to its involvement in the metabolism of opioids and other similar drugs. Indeed, it plays a pivotal role in the metabolism of endocannabinoid arachidonoylethanolamide (anandamide), 20-hydroxyeicosatetraenoic acid ethanolamide (20-HETE-EA), and 8,9-, 11,12-, and 14,15-epoxyeicosatrienoic acid ethanolamides (EpETrE-EAs), potentially modulating the endocannabinoid system signaling [60,61]. It is well described that the polymorphisms of this gene may influence the metabolism of fatty acids, steroids, and retinoids [62,63,64,65]. Moreover, its involvement in the oxidative metabolism of drugs, such as adrenoceptor antagonists, antiarrhythmics, and tricyclic antidepressants, has been demonstrated [60,61,66].

The CYP2D6 gene, as all enzymes belonging to the CYP class, is responsible for the hepatic phase I metabolism of foreign substances and drugs [67]. The P450 family convert drugs into electrophilic intermediates, which are then conjugated by phase II enzymes (e.g., UDP glucuronosyltransferase and N-acetyltransferase), and finally excreted. Although at least 60 P450 genes exist, the most studied subsets are CYP1A2, CYP2A6, CYP2B6, CYP2C19, CYP2D6, CYP2E1, and CYP3A4, which are responsible for the metabolism of the vast majority of prescription and over-the-counter drugs [68].

Considering this evidence, this enzyme is of medico-legal relevance. In addition, many antidepressants (e.g., nortriptyline) can also increase their toxicity and cause severe side effects in the presence of rapid or ultra-rapid metabolisers of CYP2D6. Therefore, understanding the CYP2D6 haplotype information of all metabolizer phenotypes can be very relevant to the forensic community [69,70].

Indeed, Koren et al. [43] and Koski et al. [25] performed targeted CYP2D6 genotyping of some deaths to investigate the cause and/or modalities of death in a number of medico-legal cases. Furthermore, the case described by Koren et al. [43] of CYP2D6 genotyping was a classic example of the ultra-rapid metabolism of codeine to morphine, resulting in increased toxicity and in the subject’s death.

The study of CYP enzyme cytochromes plays a fundamental role in these cases. Furthermore, these enzymes are not only expressed in the liver, but also in other cells, such as those of the central nervous system (CNS) [71,72,73].

These results correspond to those discussed in this systematic review: as summarized in Table 2, the CYP2C19 and CYP3A4 genes are frequently investigated in order to determine the possible influence of the genetic predisposition and the cause of death. Indeed, Shen M. et al. [28], in a study of 300 healthy individuals that were administered Zolpidem, affirmed that the *18 CYP3A4 and *2 CYP2C19 alleles were associated with poor metabolism, increasing the drug’s toxicity. Genetic factors play a crucial role in drug metabolism with important implications in forensic toxicology. Ciszkowski C. [29], in a case study about the death of a healthy 2-year-old boy after codeine and acetaminophen administration, reported that cytochrome P-450 2D6 (CYP2D6) was genotyped and revealed a functional duplication of the CYP2D6 allele, resulting in an ultrafast metabolizer phenotype. A similar result was published by Koren G. et al. [43], concerning sudden death after codeine administration due to the genetic polymorphism of CYP2D6. The codeine prescribed was within the recommended range; however, the increased conversion of codeine to morphine resulted in the toxic buildup of morphine. In another study [34], a 52-year-old female died after the administration of doxepin (Tricyclic Antidepressants); the analysis of blood through LC–MS, allowed the authors to state that the death of the woman was due to a cytochrome polymorphism and an interaction with other drugs, rather than an overdose. Furthermore, Wu A.H. [40] reported that, in a case report of a young woman who took codeine and caused a car accident, the pharmacogenetic study allowed them to affirm that she was not in a state of acute intoxication, thus this element provided evidence for her release from jail. Deaths related to venlafaxine administration and due to rapid drug metabolism with increased toxicity and patient death have also been reported [44,50].

P-gp is another protein involved in the functionality of drugs that affect the CNS, especially psychotropic drugs. These drugs not only bind to the receptor, such as the dopamine D2 receptor (D2-R), but also to P-gp and other metabolic enzymes, and is encoded by the MDR1 (or ABCB1) gene. In particular, P-gp is a 170 kDa ATP-dependent drug transporter and plays an important role in protecting the brain from potentially harmful substances [74,75]. Interindividual and population differences play a decisive role in the functionality of endogenous toxins, psychotropic drugs, and xenobiotics [76].

As explained in the present systematic review, Neuvonen A.M. [47] analyzed 112 deaths following the administration of digoxin, and the pharmacogenetic study using quantitative real-time PCR showed a link between ABCB1 polymorphisms and increased mortality, suggesting genotyping analysis prior to digoxin treatment. However, another study [49] did not report an increased risk of mortality after the MDR1gene pharmacogenetic study of P-gp after methadone administration. Further studies are needed to evaluate the importance of P-gp in forensic toxicology.

Another protein studied in pharmacogenetics is the p11 protein (also called S100A10), which plays a key role in the dynamic modulation of serotonin and has been implicated in major depressive disorder (MDD) and in the activity of antidepressant drugs. Some p11 genotypes have been shown to respond less to antidepressant therapy, causing increased suicides [77]. However, in the present systematic review, there are no studies evaluating the importance of this protein in forensic toxicology; future toxicological studies can clarify this aspect in cases of suicides.

According to Budowle et al. [78], the analysis of genetic variation and its effects on metabolism should be applied in sudden, unexplained suicides or deaths associated with chronic or acute drug therapy, even in sudden cardiac deaths [79]. Agrawal et al. [80], in a recent review, show that pharmacogenetics, through the study of gene polymorphisms, is a very useful tool in toxicology. Gene variants of enzymes that metabolize or transport drugs change the bioavailability and the therapeutic/toxic dose of the drug. In forensic toxicology, whole blood sampling is preferred for the pharmacogenetic study. Furthermore, chronic drug intake should also be considered in these cases, as there is an impact provided by acquired tolerance to drugs. Therefore, a person’s death can result from even very low concentrations of a drug, since they have not developed tolerance to it [81].

Sajantila et al. [82] argue that pharmacogenetics has great implications in malpractice claims cases. A doctor, in fact, could administer a drug during the hospitalization of a patient in a dosage considered normal; however, that drug may either not have the intended therapeutic effect or be harmful and cause death. It is not possible to use pharmacogenetics alone, but only in addition to the data of the crime scene investigation, pathology, and forensic histopathology. The correlation and the multidisciplinary approach guarantee with certainty the cause of death and the evaluation of the behavior of healthcare professionals in cases of malpractice claims [83].

A recent study [84] declared that, in the future, molecular autopsy and pharmacogenetics will be increasingly important and will play a key role in determining drug-related and sudden deaths.

**Figure 2:**
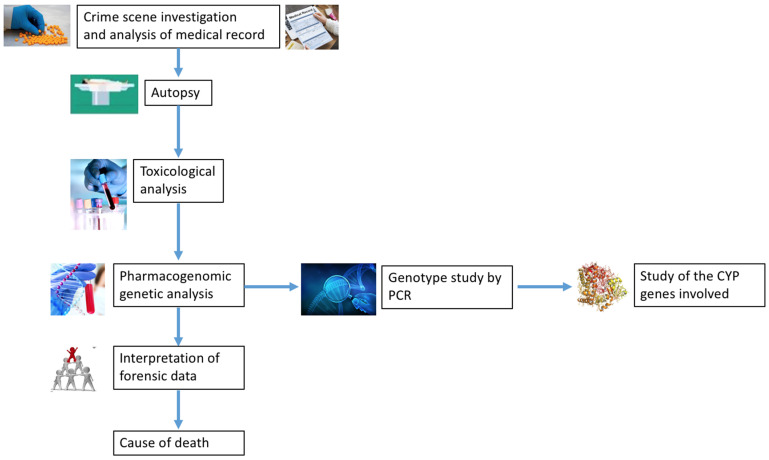
Investigative protocol for cases of suspected death related to the administration of drugs or abuse of substances. It is necessary to perform a DNA investigation, through a PCR test, analyzing the CYPs involved for the substance suspected of having been administered.

The present study highlights the importance of forensic pharmacogenetics, a field of toxicology still not fully understood, which is of great help in cases of sudden death, overdose-related deaths, deaths after the administration of a drug, and also in cases of malpractice claims. In fact, to date, this genetic investigation is not routinely used in these types of death and leads the forensic pathologist to make mistakes in the establishing of the cause of death [85,86,87]. Therefore, the purpose of this study is to provide the forensic community with a workflow to follow in cases of deaths related to the administration of drugs or substances of abuse (Figure 2). For the study of forensic pharmacogenetics, it is desirable that the forensic unit have a genetics laboratory, allowing for a complete genetic investigation [88]. Obviously, the costs of the analysis depend on the updating of techniques; those of the latest generations have a higher cost [89]. For example, Stamer et al. [90] in 2002 showed in their study that genotyping by real-time PCR with hybridization probes produced more reliable results than conventional PCR, but at much higher costs. Furthermore, according to Muller et al. [91], the real-time long-PCR analysis provides cytochrome genotyping results in 1 working day with a practical time of 3–4 h. A 2012 review [92] concluded that real-time PCR should be used routinely in these kinds of analyses, considering the easy availability of this molecular analysis and also the low cost. An original article from 2010 confirmed that PCR gave reliable and low-cost results in forensic applications [93]. To date, more recent studies have shown that forensic genetics should always be used routinely in forensic practice [94,95]. Molecular techniques, such as PCR, are consolidated and widespread in the forensic field. Particularly molecular autopsy is an indispensable tool for the pathologist in order to define the cause of death. Usage times are also much shorter with reliable results in approximately one day. These aspects should encourage the forensic pathologist to always introduce these molecular techniques, such as PCR, now well-known not only in the microbiology field [96,97,98,99,100].

## 5. Conclusions

To date, drug-related deaths are very common and new drugs are emerging [101,102,103,104]. However, there is an increase in deaths also related to the use of other commonly used drugs used for chronic diseases (antidepressants, antipsychotics, and non-steroidal anti-inflammatory NSAIDs). These deaths not only occur at home, but also in hospital, and are often related to complaints of malpractice by relatives [105,106,107,108,109]. In this scenario, these new techniques, posed at the center between toxicology and genetics, allow the forensic team to reach a certain diagnosis of the cause of death and not to make mistakes [110,111,112]. In the same manner, further studies should be conducted in order to identify new molecular biomarkers that could be useful both for forensic purposes, such as the identification of the cause of death, and for the identification of abuse drugs [113,114,115].

Based on the results of this systematic review, in the forensic field, gene investigation should be performed in relation to the hypothesis of the drug assumption: the most investigated genes are CYP2C19, CYP2D6, and CYP3A4.

The application of pharmacogenetics has important repercussions from both medical-legal and ethical points of view. The management of informed consent and the DNA database collection represent important concerns that should be considered before the introduction of the genetic unit in a forensic institute. Moreover, one of the most important problems is the informativeness of genetic testing, which could raise issues related to uncertain paternity. In this way, relatives may not consent to pharmacogenetic tests being carried out, and this may have repercussions on establishing the truth. Considering the application of pharmacogenetics to forensic science, it will be necessary to take into account these considerations both in practical applications and in the research field.

In the same manner, the multidisciplinary approach is a well-established approach in the forensic field that has to enter into the routine practice of the forensic field. This is highlighted by this systematic review by providing the forensic team with a workflow to use in cases of suspected drug-related death.

## Figures and Tables

**Table 1:** Main enzymes involved in drug metabolism in forensic toxicology.

Drugs of Toxicological Interest and Their Metabolizing Enzymes [22,23,24]	
Gene Involved in Drug Metabolization	Drugs Metabolized
*CYP2C19*	Amitriptylineim, Ipramine, Diazepam, Citalopram, Carisoprodol, several Anticonvulsants
*CYP2D6*	Amphetamines, Codeine, Oxycodone, Hydrocodone, Methadone, Tramadol, some Antipsychotics, some Tricyclic Antidepressants, Steroids
*CYP3A4*	Benzodiazepines, Fentanyl, Methadone, Buprenorphine, Hypnotics, some Antipsychotics, some Tricyclic Antidepressants
*CYP2E1*	Ethanol, some Anaesthetics, Paracetamol, Verapamil

**Table 2:** Summary of the details of the systematic review.

Reference	Study Design	Number of Cases	Type of Drugs	Type of Sample	Toxicological Method	Genotypic Method	Gene Study	Main Findings about the Genetic Investigation in Order to Define the Cause of Death (* Indicates the Haplotype)
Kupiec T.C. [20]	Case report	Death of a 30-year-old male	Methadone and benzodiazepines	Blood	LC–MS	Quantitative real-time PCR	CYP3A4 (benzodiazepines)- CYP2D6 (Methadone)	The subject had a CYP2D6 polymorphism that increased its toxicity combined with benzodiazepines interaction resulting in death.
Koski A. [25]	Original article	11 deceased people	Doxepin (tricyclic antidepressants)	Blood	GC–MS	Quantitative real-time PCR	CYP2D6	In cases of indeterminate death after drug administration, cytochrome genotyping is a very useful method for establishing the cause of death. Completely non-functional CYP2D6 cytochromes (* 3/* 4) with poor drug metabolism were found in the present study.
Chiurillo M.A [26]	Original article	110 healthy individuals	EDTA	Blood	GC–MS	PCR tetra-primer	CYP2D6	Analysis of CYP2D6 * 3, * 4, * 5, * 6, and 10 * alleles of 110 Velezuelan, unrelated healthy individuals. Allele frequencies obtained for * 4 (15.9%) and * 10 (16.4%) had a narrow therapeutic window and showed increased toxicity.
Shen M. [27]	Original article	300 healthy individuals	Zolpidem (sedative and hypnotic drug)	Blood	UPLC–MS/MS	PCR	CYP2D6, CYP2C19, CYP2C9, CYP3A4, CYP1A2	The *18 CYP3A4 and *2 CYP2C19 alleles are associated with poor Zolpidem metabolism with increased toxicity. Genetic factors play a crucial role in drug metabolism with implications in forensic toxicology.
Lam J. [28]	Original article	68 deceased people	Codeine	Blood	GC–MS	PCR- QiaSymphony DNA purification system	CYP2D6	Individuals carrying the ABCB1 1236T variant of CYP2D6 had higher codeine concentrations than the general population with increased toxicity.
Ciszkowski C. [29]	Case report	Death of a healthy 2-year-old boy	Codeine, Acetaminophen	Blood	GC–MS	PCR	CYP2D6	In the present case, cytochrome P-450 2D6 (CYP2D6) was genotyped and revealed the functional duplication of the CYP2D6 allele, resulting in an ultrafast metabolizer phenotype. The codeine prescribed was within the recommended range; however, the increased conversion of codeine to morphine resulted in a toxic buildup of morphine.
Høiseth G. [30]	Original article	75 Healthy individuals	Carisoprodol (muscle relaxant)	Blood	GC–MS	TaqMan-based realtime PCR assays	CYP2C19	This study found no evidence of an association between CYP2C19 genetics and the mortality risk of carisoprodol.
Thieme D. [31]	Original article	23 Healthy individuals	Amitriptyline, Nortriptyline (Tricyclic Antidepressant)	Hair	LC–MS	TaqMan-based realtime PCR assays	CYP2D6, CYP2C19	The genotypes of CYP2C19 (alleles *2, *3, and *4) and CYP2D6 (*3, *4, and *6) were examined and a substantial change in metabolites was seen from the wild-type variant.
Koski A. [32]	Original article	202 deceased people	Amitriptyline (Tricyclic Antidepressant)	Blood	LC–MS	Long PCR reactions	CYP2D6, CYP2C19	Pharmacogenetic analysis of CYP2D6 and CYP2C19 genotypes is of great help in cases of subjects who die after drug administration.
Tawfik Khattab A.M. [33]	Original article	187 Healthy individuals	Organophosphorus drugs	Blood	GC–MS	Long PCR reactions	CYP2D6, CYP2C19	In some populations, some genetic polyforms are very frequent, so it is important to consider this in pharmacogenetics.
Neukamm M.A. [34]	Case report	52-year-old female	Doxepin (Tricyclic Antidepressants)	Blood	LC–MS	PCR	CYP2D6	The death of the woman was due to a cytochrome polymorphism and an interaction with other drugs rather than an overdose.
Madadi P [35]	Case report	Child aged 5 years 9 months	Clarithromycin (antibiotic) and valproic acid (antiepileptic)	Blood	GC	TaqMan-based realtime PCR assays	CYP2D6	The child had a particular genetic polymorphism of cytochrome CYP2D6 (CYP2D6 * 2A/* 41). Co-administration of clarithromycin and valproic acid prevented drug elimination and increased bioavailability. It is important to analyze pharmacogenetics, pharmacokinetics, and drug interactions in similar cases in this case.
Launiainen T. [36]	Original article	123 deceased people	Venlafaxine (serotonin and norepinephrine reuptake inhibitors—SSNRIs)	Blood	GC–MS	Long PCR reactions	CYP2D6	This study found no evidence of an association between CYP2D6 genetics and the mortality risk of venlafaxine.
Riccardi L.N. [37]	Original article	32 deceased people	Central nervous system drugs	8 post-mortem saliva, 8 blood, 5 spleen samples, and 10 buccal swabs from a population study, and 1 paraffine-embedded tissue	GC–MS	Tetraplex PCR	CYP2D6	Excellent method for evaluating CYP2D6 and its implications in the forensic field.
Jakobsson G. [38]	Original article	174 deceased people	Oxycodone (opioid)	Blood	LC–MS	Digital droplet PCR	CYP2D6	In toxicology, not only the concentration of metabolites in the blood is important to assess drug toxicity, but also the genotyping of cytochromes. A fast or slow phenotype changes the toxicity of a drug. A pharmacogenetic study should always be performed.
Vevelstad M. [39]	Original article	29 deceased people	Paramethoxymethamphetamine (PMMA)	Blood	LC–MS	Quantitative real-time PCR	CYP2D6	In some, CYP2D6 concentrations of PMMA were higher than in others, with metabolites at a lower blood concentration.
Wu A.H. [40]	Case report	Young woman	Codeine	Blood	LC–MS	Quantitative real-time PCR	CYP2D6, UGT2B7, CYP3A4	In this case report, a young woman had taken codeine and caused a car accident. The young woman initially went to jail. However, through a pharmacogenetic study, it was possible to state that she was not in a state of acute intoxication so this element provided evidence for her release.
Bastami S. [41]	Original article	20 Healthy individuals	Tramadol (opioid)	Blood	HPLC	Quantitative real-time PCR	CYP2D6	The study of the CYP2D6 genotype helps to establish with greater certainty the metabolic relationship of tramadol with its metabolite to evaluate the time of drug intake.
Levo A. [42]	Original article	33 deceased people (11 males and 22 females)	Tramadol (opioid)	Blood	LC–MS	PCR	CYP2D6	The genetic variation of drug metabolizing enzymes is substantial and can be studied in forensic toxicology. Furthermore, genetic factors play a dominant role in the metabolism of individual drugs.
Koren G. [43]	Case report	Male infant	Codeine and Paracetamol	Blood	GC–MS	PCR	CYP2D6	The CYP2D6 *2A allele with CYP2D6 *2 × 2 gene duplication (ultra-rapid metaboliser) resulted in increased morphine formation from codeine, resulting in drowsiness and the death of the child.
Piatkov I. [44]	Original article	10 suicide cases	Venlafaxine (selective serotonin and norepinephrine reuptake inhibitors-SSNRIs)	Blood	GC–MS	Quantitative real-time PCR	CYP2D6, CYP2C19, CYP2C9	Venlafaxine, which caused neurotoxicity and suicide in these people, was related to the particular functional genetic polymorphisms of cytochrome P450, especially CYP2C19 *17.
Rahikainen A.L. [45]	Original article	349 suicide cases	Citalopram (antidepressant of the selective serotonin reuptake inhibitor class)	Blood,	LC–MS	PCR	P-gp	Genetic variation in efflux transporter and permeability glycoprotein (P-gp) in women taking citalopram was associated with completed violent suicides and even violent suicide attempts.
Drevin G. [46]	Case report	35-year-old man	Ethanol, Morphine, Antidepressants	Blood	GC–MS	Taqman real-time PCR analyses	CYP2D6, CYP2C19	Cytochrome genotyping is a very useful method for establishing the cause of death.
Neuvonen A.M. [47]	Original article	112 deceased people	Digoxin (heart failure)	Blood	HPLC	Quantitative real-time PCR	P-gp ABCB1gene	There is a link between ABCB1 polymorphisms and increased mortality, suggesting genotyping analysis prior to digoxin treatment.
Fonseca S. [48]	Original article	100 deceased people	Tramadol (opioid)	Blood	GC–MS	Quantitative real-time PCR	CYP2D6	Poor metabolizers have very low metabolic capacity and higher metabolic ratios. The metabolism of tramadol is correlated with the phenotype of the metabolizer and it is essential to know the phenotype in cases of opioid-related deaths.
Buchard A. [49]	Original article	90 deceased people	Methadone (opioid)	Blood	GC–MS	Quantitative real-time PCR	P-gp MDR1gene	This study found no evidence of an association between P-glycoprotein MDR1 gene genetics and the mortality risk of methadone.
Karlsson L. [50]	Original article	94 deceased people	Venlafaxine (selective serotonin and norepinephrine reuptake inhibitors-SSNRIs)	Blood	GC–MS	Quantitative real-time PCR	CYP2C19, CYP2D6	The CYP2D6 genotype influences the O-demethylation of venlafaxine, while CYP2C19 influences the N-demethylation of venlafaxine, which affects the toxicity of this drug.
Andresen H. [51]	Original article	11 deceased people	Morphine and codeine	Blood	GC–MS	Quantitative real-time PCR	CYP2D6	Pharmacogenetic evaluation in forensic toxicology is essential, as the genetic polymorphisms of cytochromes play a key role in drug toxicity.
Jannetto P.J. [52]	Original article	25 deceased people	Fentanyl	Blood	GC–MS	Quantitative real-time PCR	CYP3A4, CYP3A5	Post-mortem data are evidence that CYP3A4 and CYP3A5 are involved in the metabolism of fentanyl. In particular, homozygous CYP3AS * 3 causes an altered metabolism of fentanyl with an increase in its concentration in the blood. Pharmacogenetics and molecular autopsy are crucial in these deaths.

## Data Availability

Data sharing is not applicable; no new data were created or analyzed in this study.
